# A Comparative Study of Surgical Correction of Idiopathic Scoliosis With Spinal Transpedicular Metal Structures in Children

**DOI:** 10.3389/fped.2022.871117

**Published:** 2022-05-16

**Authors:** Nurbek Nadirov, Sergey Vissarianov

**Affiliations:** ^1^Mother and Child Health Center, Department of Orthopedics, University Medical Center, Nur-Sultan, Kazakhstan; ^2^H.Turner National Medical Research Center for Children's Orthopedics and Trauma Surgery of the Ministry of Health of the Russian Federation, Saint Petersburg, Russia

**Keywords:** idiopathic scoliosis, children, surgical treatment, transpedicular metal structures, spinal system

## Abstract

A comparative study of surgical correction of idiopathic thoracic scoliosis using transpedicular spinal systems in children was performed. The study showed that using the transpedicular supporting elements along the entire length of the deformation (concave and convex sides) using the VCM (vertebral column manipulation) system, the correction was significantly better (*p* ≤ 0.05) than for the patients for whom the screws were not installed over two or more vertebrae from the concave side of the curvature, regardless of the magnitude of the spinal deformity. The kyphosis and lordosis were completely restored to their physiological values in all groups of patients.

## Introduction

Surgical treatment of severe forms of thoracic idiopathic scoliosis remains an urgent and not fully resolved problem. Some surgeons perform surgical intervention exclusively with dorsal approach, using constructions with a large number of supporting elements (1–3), others use ventral systems to correct the deformity (4–6). A number of authors describe combined interventions from anterior and posterior sides of the spine, when dealing with idiopathic scoliosis (1, 7). When correcting idiopathic scoliosis, it is important to restore the frontal and sagittal profiles of the deformed spine, rotate the apical vertebra and maintain this result in the postoperative period (8). Incorrect preoperative planning when choosing the level and length of fixation often leads to violation of the sagittal profile of the spinal column, which could lead to the development of kyphosis and degenerative processes (9).

In recent years, there is a tendency to use transpendicular spine fixation for the correction of spinal deformity in patients with idiopathic scoliosis (10). The preference toward this type of metal structures is explained by certain features and advantages over other systems. In the available studies, it has been proven that this system allows to achieve significant correction of deformity in all planes, stable fixation in the postoperative period, as well as limits the needs for extended instrumentation compared to the hook spinal systems (8, 11). In addition, transpedicular multi-support hardware allows to prevent long-term loss of correction and deformity progression.

However, there is a discrepancy between different researchers regarding the results of such surgeries using multi-support systems with transpedicular support elements for patients with idiopathic scoliosis. Some authors claim that when correcting the curvature of the spine in patients diagnosed with thoracic idiopathic scoliosis, hybrid spinal systems are not inferior to transpedicular metal structures (12). Others prove the advantages and effectiveness of multi-support spinal metal structures with transpedicular supporting elements in comparison with hybrid systems (13, 14). A number of surgeons note that when correcting spinal deformity with laminar and hybrid metal structures, restoration of the sagittal profile of the thoracic spine reaches physiologically correct values compared to when transpedicular spinal systems is used. According to their data, transpedicular metal structures contribute to the flattening of the kyphosis of the thoracic region following correction (15).

### Aim

The aim of this study is to conduct a comparative analysis of the results of surgical correction of spinal deformity in children diagnosed with thoracic idiopathic scoliosis using transpedicular systems using various surgical methods.

## Materials and Methods

The work is based on the analysis of the results of surgical treatment of 80 children diagnosed with thoracic idiopathic scoliosis grade 3–4: 12 (15%) male patients and 68 (85%) female patients aged 14 to 17 years. In all children, right-sided curvature was observed. The type of scoliotic deformity was determined based on the Lenke classification (16). All patients (80 patients) were of the first type (Lenke 1); the sagittal contour in most of them was marked as normokyphosis. Patients were divided into 4 groups depending on the type of surgical correction. The choice of the technique used to correct spinal deformity in children with idiopathic thoracic scoliosis was determined by the initial size of the main curvature, its mobility, and the anatomo-anthropometric features of the vertebral arch roots comprising the curvature arch.

All patients underwent X-ray of the spine in two standard projections and functional scans with lateral flexion. In order to determine the size of the vertebrae arch roots along the arch of deformity and the magnitude of rotation of the bodies of the apical vertebrae, computed tomography (CT) was performed. Spine X-ray and CT were performed both before and after the surgery in order to analyze the results of the surgical treatment. To exclude a pathology of spinal canal, an MRI was performed before the operation. Patients were monitored for the treatment effectiveness 6, 12, 18 months, and then once a year following the surgery. Statistical analysis was performed using the STATISTICA 6.0 software. When comparing pairs of groups for various characteristics in dynamics, paired Wilcoxon and Student's tests were used. When comparing independent pairs of groups, non-parametric Wilcoxon and Mann-Whitney tests were used. Samples' homogeneity was checked by the Kolmogorov-Smirnov test to confirm the normal distribution. *P* values of <0.05 were regarded as statistically significant.

The mobility of the deformity was calculated using the following formula:


$$\begin{array}{r} -M=\frac{\text{Standing scoliosis}-\text{Scoliosis with lean}}{\text{Standing scoliosis}}*100 \end{array}$$

The percentage of scoliotic deformity correction was calculated using the following formula:


$$\begin{array}{r} C=\frac{\text{Standing scoliosis before surgery}-\text{Standing scoliosis after surgery}}{\text{Standing scoliosis before surgery}}*100 \end{array}$$

The percentage of apical vertebra derotation was determined according to the following formula:


$$\begin{array}{r} DR=\frac{\text{Rotation before surgery}-\text{Rotation after surgery}}{\text{Rotation before surgery}}*100 \end{array}$$

In the first group (20 patients with 40° to 79° Coob's angle and the mobility the main curve of more than 30%), the deformity correction was performed with a multi-support transpedicular hardware only from the dorsal approach with Halo-tibial traction. In this group of patients, transpedicular supporting elements were installed along the entire length of the deformity arch and the VCM (vertebral column manipulation) system was used at the apex in order to carry out a true derotation maneuver of the vertebral bodies. In the second group (20 patients with 51° to 79° Cobb's angle and the mobility of the main curve mora than 30%), the entire surgery was also performed using dorsal approach. We failed to put two or more screws on the concave side of the curvature due to small anatomical and anthropometric dimensions of the base of the vertebral arches in this group of patients. After the installation of transpedicular support elements, Halo-tibial traction was performed and the first rod, bent along the physiological curvature, was sequentially fixed in the support elements of the metal structure along the convex side of the deformity, while simultaneously correcting the kyphotic and scoliotic components of the deformity by applying direct pressure on the apex of the main arch, translational maneuver, and segmental compression. Subsequently, the second rod, bent along the physiological curvature, was placed in the supporting elements of the metal structure on the opposite side, and the final correction was performed using segmental distraction. The operation was concluded by putting a posterior bone graft. In the third group (20 patients with 80° to 114° Cobb's angle and the mobility of the main curvature of <30%), the surgical intervention was performed using two approaches (in 13 patients at the same time and in 7 patients in two stages). Using anterolateral approach, disepiphysectomy at the apex of the main deformity arch at the 4–5 levels in combination with corporodesis using dorsal approach, spinal deformity was corrected and stabilized with a multi-support transpedicular spinal system combined with Halo-tibial traction and dorsal fusion with autologous bone. For this group of patients, pedicle screws were installed along the entire length of the main curvature, and the VCM system was used to perform a true derotation maneuver of the vertebral bodies. In the fourth group (20 patients with 80° to 148° Cobb's angle and the mobility of the main curvature of <30%), surgical intervention was performed using two approaches (in 9 patients simultaneously and in 11 patients in two stages). Using anterolateral approach, disepiphysectomy at the apex of the main deformity arch at the 4–5 levels in combination with corporodesis using dorsal approach, the curvature of the spine was corrected and stabilized with a multi-support transpedicular spinal system. We failed to install two or more screws on the concave side of the curvature due to the small size of the base of the vertebral arches. Corrective manipulations during the surgery in this group was similar to the technique used in the second group.

## Results

**Figure 1:**
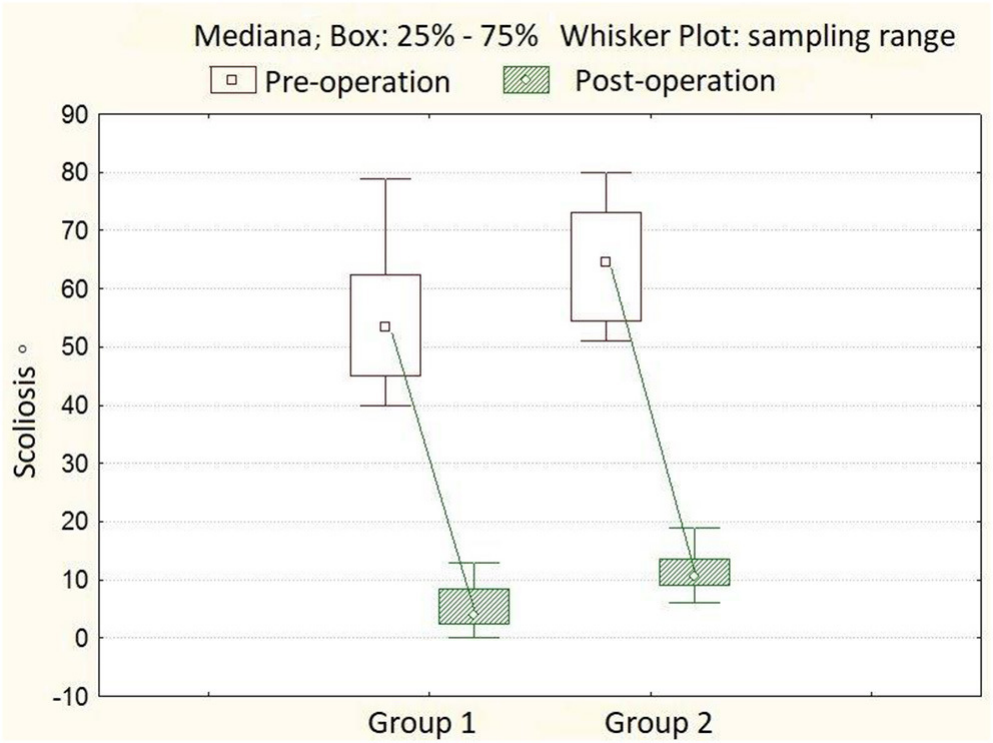
A boxplot showing the dynamics of scoliosis angles in patients from groups 1 and 2.

**Figure 2:**
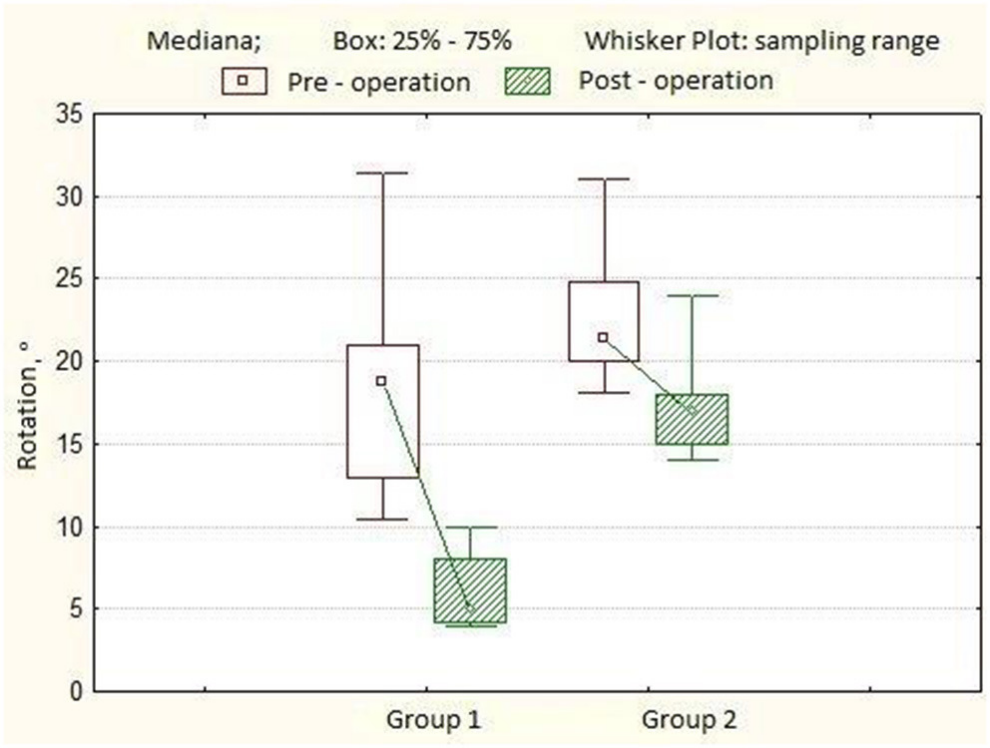
A boxplot showing the dynamics of the angles of rotation of the apical vertebra in patients from groups 1 and 2.

In the first group of patients, the angle of scoliotic deformity ranged from 40° to 79° (mean 54.8° ± 10.9°), kyphotic angle—from 4° to 50° (mean 20.3° ± 11.8°), lordosis value—from 15° to 54°, mean 31.7° ± 12.1°. The rotation of the apical vertebra ranged from 10.4° to 31.4° (mean 18.8° ± 4.2°). After surgery, the main angle of scoliotic deformity was 4° ± 3.3° (0°-13.0°), the percentage of correction was 92.1 ± 7.1%. The angle of kyphosis in the thoracic region is 20.6° ± 7.2°, the angle of lordosis in the lumbar region is 25.2° ± 6.4°. The rotation of the apical vertebra was 5.0° ± 1.3° (4.0°-10.0°), the percentage of derotation was 65.9 ± 9.5%. The length of the hardware is 11 ± 0.7 (10–12) vertebrae. The number of transpedicular support elements per patient varied from 20 to 24, with an average of 21 screws. Twelve months after the surgery, the magnitude of the scoliotic curve was 4° (0.0°-14.0°), the angle of kyphosis was 21.1° ± 6.7°, and the lordosis was 25.7° ± 6.6°. After 3 years of observation, the angle of scoliosis was 7° (0°-16°), the magnitude of kyphosis was 21.4° ± 6.5°, and the angle of lordosis was 26.9° ± 7.0° (Table 1). In the second group of patients, the angle of scoliotic deformity ranged from 51° to 79° (mean 64.4° ± 9.9°), kyphotic angle—from 7° to 40° (mean 18.9° ± 8.2°), the magnitude of lordosis from 20° to 50° on average 29, 1° ± 8.5°. Rotation of the apical vertebra—from 18.1° to 31.0° (mean 21.4° ± 2.6°). After the operation, the angle of scoliotic deformity of the spine was 10.5° ± 3.1° (6.0°-19.0°); the percentage of correction is 81.8 ± 6.3% (Figure 1). The angle of kyphosis in the thoracic region is 23.4° ± 5.1°, the angle of lordosis in the lumbar region is 28.2° ± 3.8°. The rotation of the apical vertebra was 17.0° ± 1.9° (14.0°-24.0°), the percentage of derotation was 23.2 ± 4.3% (Figure 2). The length of the metal structure is 11 ± 0.7 (10–12), vertebrae. The number of supporting elements per patient varied from 15 to 22, with an average of 18 screws. Twelve months after surgery, the magnitude of the scoliotic curve was 11.5° (7.0°-20.0°), the angle of kyphosis was 23.0° ± 7.0°, and the angle of lordosis was 27.3° ± 5.4°.After 3 years of observation, the angle of scoliosis was 13.0° (8.0°-23.0°), the magnitude of kyphosis was 21.9° ± 6.6°, and the angle of lordosis was 27.7° ± 5.6° (Table 2).

**Table 1:** The main descriptive statistics for the indicators of the first group, *n* = 20.

	**Before surgery**	**After surgery**	**One year**	**Three years**
			**after surgery**	**after surgery**
Scoliosis angle in standing position, °	54.8 ± 10.9	4.0 [0.0; 13.0]	4.0 [0.0; 14.0]	7.0 [0.0; 16.0]
Scoliosis angle in tilt position, °	33.9 ± 11.6			
Mobility, %	36.7 [18.2; 63.8]			
Apical vertebral rotation, °	18.8 [10.4; 31.4]	5.0 [4.0; 10.0]		
Angle of kyphosis, °	20.3 ± 11.8	20.6 ± 7.2	21.1 ± 6.7	21.4 ± 6.5
Angle of lordosis, °	31.7 ± 12.1	25.2 ± 6.4	25.7 ± 6.6	26.9 ± 7.0
Scoliosis correction, %		92.1 ± 7.1	91.2 ± 6.3	90.0 ± 8.5
Apical vertebral derotation, %		65.9 ± 9.5		
Length of the metal structure, vertebrae (spondylodesis area)	11 ± 0.7			

**Table 2:** The main descriptive statistics for the indicators of the second group, *n* = 20.

	**Before surgery**	**After surgery**	**One year**	**Three years**
			**after surgery**	**after surgery**
Scoliosis angle in standing position, °	64.4 ± 9.9	10.5 [6.0; 19.0]	11.5 [7.0; 20.0]	13.0 [8.0; 23.0]
Scoliosis angle in tilt position, °	42.0 ± 9.2			
Mobility, %	35.2 [17.8; 48.5]			
Apical vertebral rotation, °	21.4 [18.1; 31.0]	17.0 [14.0; 24.0]		
Angle of kyphosis, °	18.9 ± 8.2	23.4 ± 5.1	23.0 ± 7.0	21.9 ± 6.6
Angle of lordosis, °	29.1 ± 8.5	28.2 ± 3.8	27.3 ± 5.4	27.7 ± 5.6
Scoliosis correction, %		81.8 ± 6.3	80.1 ± 6.6	78.2 ± 7.0
Apical vertebral derotation, %		23.2 ± 4.3		
Length of the metal structure, vertebrae	11 ± 0.7			

**Figure 3:**
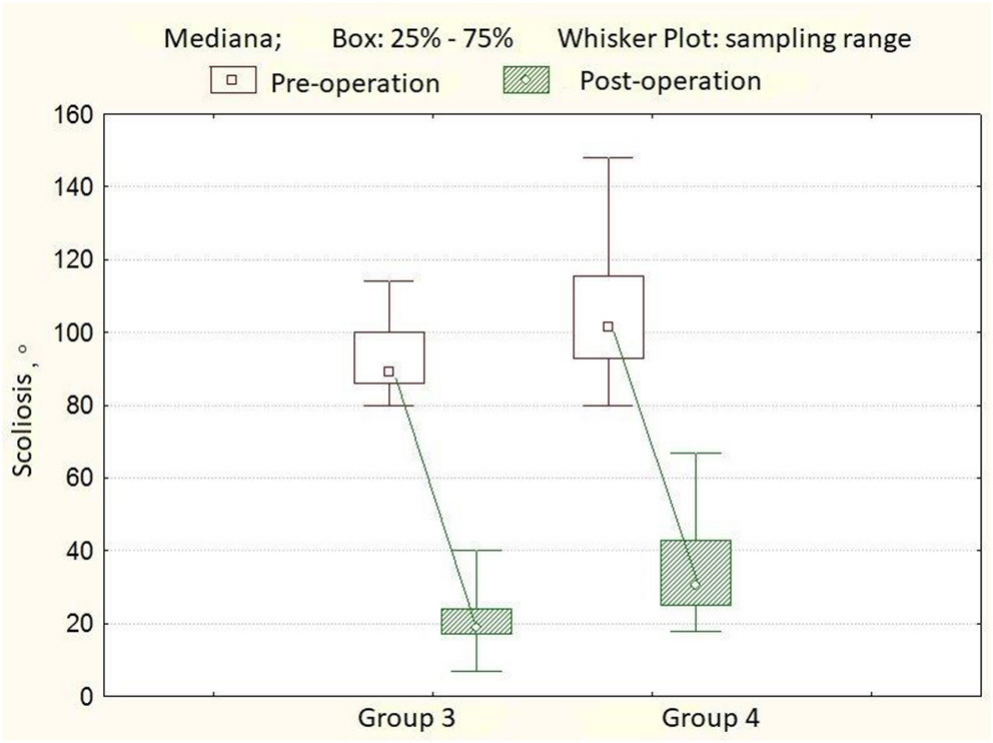
A boxplot showing the dynamics of scoliosis angles in patients from groups 3 and 4.

**Figure 4:**
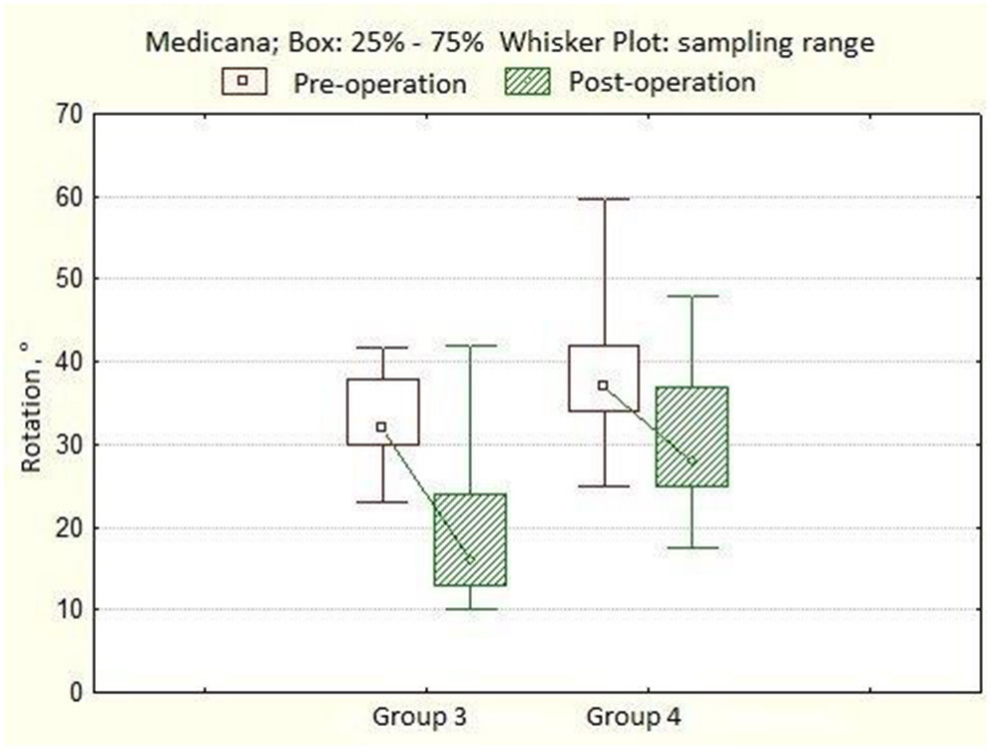
A boxplot showing the dynamics of the angles of rotation of the apical vertebra in patients from groups 1 and 2.

In the third group of patients, the angle of scoliotic deformity ranged from 80° to 114° (mean 92.6° ± 10.0°), kyphotic angle—from 15° to 66° (mean 37.2° ± 15.4°), lordosis value—from 10° to 57°, mean 36.9° ± 12.7°. Rotation of the apical vertebra ranged from 23.0° to 41.8° (mean 32.0° ± 4.0°). After surgery, the angle of scoliotic deformity of the spine was 19.0° ± 6.2° (7.0°-40.0°), the percentage of correction was 78.1 ± 7.7%. The angle of kyphosis in the thoracic region is 28.0° ± 7.1°, the angle of lordosis in the lumbar region is 32.5° ± 8.3°. Rotation of the apical vertebra 16.0° ± 4.9° (10.0°-42.0°), the percentage of derotation is 46.3 ± 11.9%. The length of the metal structure is 12 ± 0.7 (10–13), vertebrae. The number of supporting elements per patient varied from 20 to 26, with an average of 24 screws. Twelve months after surgery, the magnitude of the scoliotic curve was 21.0° (10.0°-38.0°), the angle of kyphosis was 26.5° ± 10.6°, and the angle of lordosis was 32.3° ± 10.8°. After 3 years of observation, the angle of scoliosis was 23.0° (10.0°-42.0°), the magnitude of kyphosis was 26.8° ± 9.5°, and the angle of lordosis was 31.6° ± 11.2° (Table 3). In the fourth group of patients, the angle of scoliotic deformity ranged from 80° to 148° (mean 104.5° ± 18.1°), kyphotic angle—from 10° to 92° (mean 43.3° ± 24.3°), the magnitude of lordosis—from 13° to 74°, mean of 40.8° ± 15.4°. Rotation of the apical vertebra ranged from 25° to 59.7° (mean 37.0° ± 9.0°). After surgery, the angle of scoliotic deformity of the spine was 30.5° ± 10.3° (18.0°-67.0°), the percentage of correction was 71.7 ± 17.4% (Figure 3). The angle of kyphosis in the thoracic region is 30.2° ± 8.5°, the angle of lordosis in the lumbar region is 28.3° ± 6.5°. The rotation of the apical vertebra was 28.0 ± 4.7° (17.4°-48.0°), the percentage of derotation was 22.7 ± 8.8% (Figure 4). The length of the metal structure is 13 ± 0.6 (11–14), vertebrae. The number of supporting elements per patient varied from 18 to 25, with an average of 20 screws. Twelve months after surgery, the magnitude of the scoliotic curve was 36.5° (20.0°-72.0°), the angle of kyphosis was 29.2° ± 8.6°, and the angle of lordosis was 26.8° ± 7.4°. After 3 years of observation, the angle of scoliosis was 39.0° (20.0°-75.0°), the magnitude of kyphosis was 29.9° ± 9.5°, and the angle of lordosis was 27.4° ± 8.0° (Table 4).

**Table 3:** The main descriptive statistics for the indicators of the third group, *n* = 20.

	**Before surgery**	**After surgery**	**One year**	**Three years**
			**after surgery**	**after surgery**
Scoliosis angle in standing position, °	92.6 ± 10.0	19.0 [7.0; 40.0]	21.0 [10.0; 38.0]	23.0 [10.0; 42.0]
Scoliosis angle in tilt position, °	80.3 ± 13.6			
Mobility, %	15.0 [3.5; 22.5]			
Apical vertebral rotation, °	32.0 [23.0; 41.8]	16.0 [10.0; 42.0]		
Angle of kyphosis, °	37.2 ± 15.4	28.0 ± 7.1	26.5 ± 10.6	26.8 ± 9.5
Angle of lordosis, °	36.9 ± 12.7	32.5 ± 8.3	32.3 ± 10.8	31.6 ± 11.2
Scoliosis correction, %		78.1 ± 7.7	76.3 ± 7.2	74.8 ± 7.5
Apical vertebral derotation, %		46.3 ± 11.9		
Length of the metal structure, vertebrae	12 ± 0.7			

**Table 4:** The main descriptive statistics for the indicators of the fourth group, *n* = 20.

	**Before surgery**	**After surgery**	**One year**	**Three years**
			**after surgery**	**after surgery**
Scoliosis angle in standing position, °	104.5 ± 18.1	30.5 [18.0; 67.0]	36.5 [20.0; 72.0]	39.0 [20.0; 75.0]
Scoliosis angle in tilt position, °	94.7 ± 19.4			
Mobility, %	8.4 [0.0; 29.4]			
Apical vertebral rotation, °	37.0 [25.0; 59.7]	28.0 [17.4; 48.0]		
Angle of kyphosis, °	43.3 ± 24.3	30.2 ± 8.5	29.2 ± 8.6	29.9 ± 9.5
Angle of lordosis, °	40.8 ± 15.4	28.3 ± 6.5	26.8 ± 7.4	27.4 ± 8.0
Scoliosis correction, %		71.7 ± 17.4	69.8 ± 18.5	67.6 ± 9.4
Apical vertebral derotation, %		22.7 ± 8.8		
Length of the metal structure, vertebrae	13 ± 0.6			

The percentage of the deformity correction using transpendicular spinal systems in our study is quite high in all groups of patients and the long-term angle loss is insignificant (from 0 to 4%).

## Discussion

When comparing the effectiveness of surgical correction between the first and second groups, the correction of scoliotic deformity of the spine in patients of the first group (92.1 ± 7.1%) was greater than in the second group (81.8 ± 6.3%). This can be explained by the use of two supporting elements placed in each vertebra (flexed and convex sides) included in the scoliotic arch. At the same time, it should be emphasized that the magnitude of derotation in the first group (65.9 ± 9.5%) is significantly higher compared to patients in the second group (23.2 ± 4.3%). This result is explained by the use of VCM system to achieve a true derotation of the vertebrae at the apex of the main curvature in the first group. The magnitude of the correction of kyphosis and lordosis in both groups was the same, and clinically and radiographically improvement or complete recovery was noted. Following mobilizing interventions on the anterior parts of the vertebral bodies of the main deformity curve from the anterolateral approach and dorsal correction with a metal structure with transpedicular supporting elements, it was noted that the magnitude of the correction of scoliotic deformity of the spine in the third group of patients (78.1 ± 7.7%) was greater than in the fourth group (71.7 ± 17.4%), which could be explained by the fact that installed transpedicular screws on both sides of the deformed area increase the effect on the spinal column. It should also be emphasized that the percentage of derotation of the apical vertebra in patients of the third group (46.3 ± 11.9%) was significantly higher than in the fourth group (22.7 ± 8.8%). This result was explained by the use of the VCM system to achieve a true derotation of the vertebrae at the apex of the main curvature in the third group.

## Conclusion

**Figure 5:**
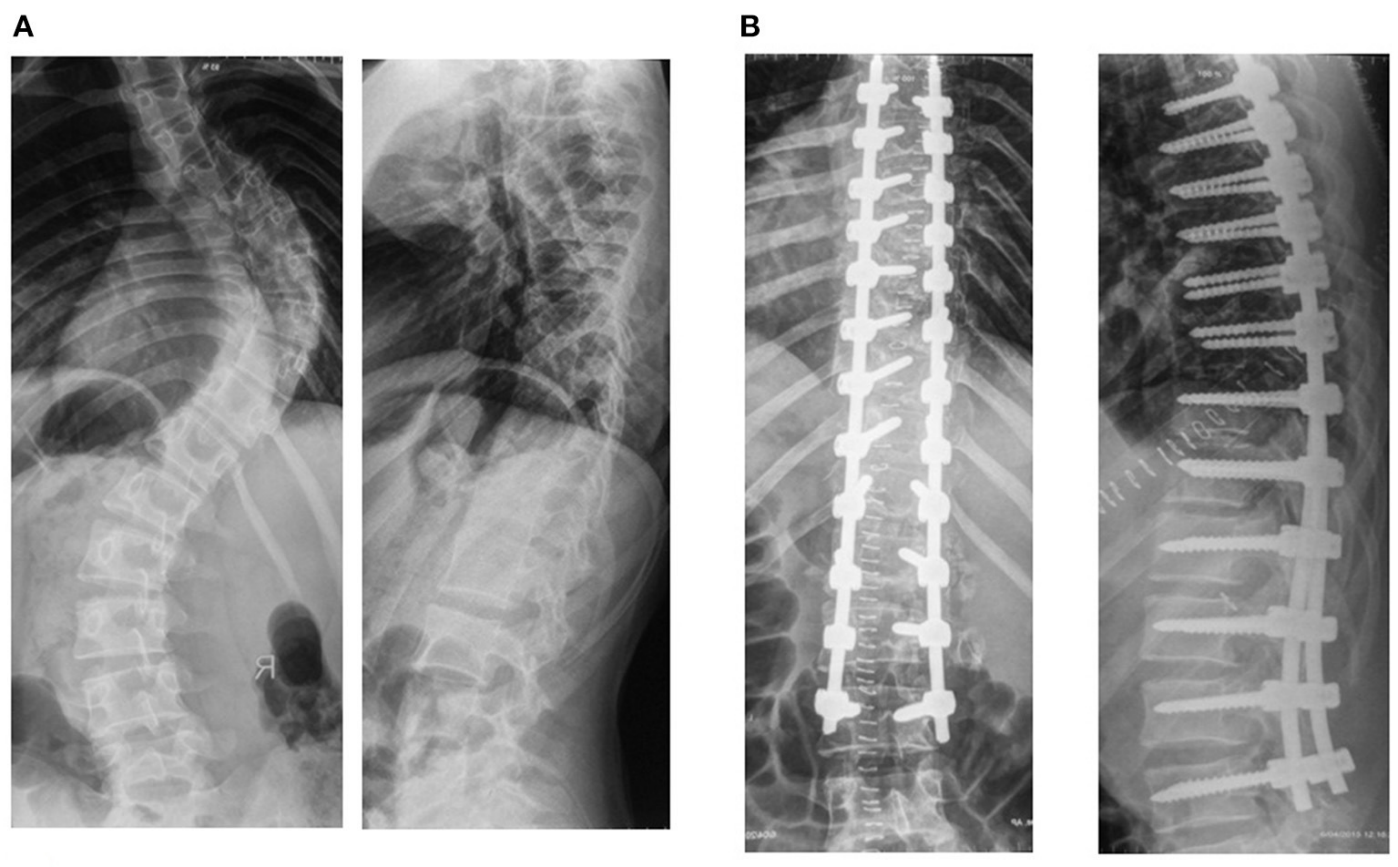
Spinal radiographs of patient T (16 years old). Idiopathic right thoracic scoliosis. **(A)** Preoperatively, the angle of scoliotic deformity was 86° according to Cobb, the angle of kyphosis was 13°, and the angle of lordosis was 22°. **(B)** Postoperatively, the angle of scoliosis was 5°, the angle of kyphosis was 22°, and the angle of lordosis was 26°.

**Figure 6:**
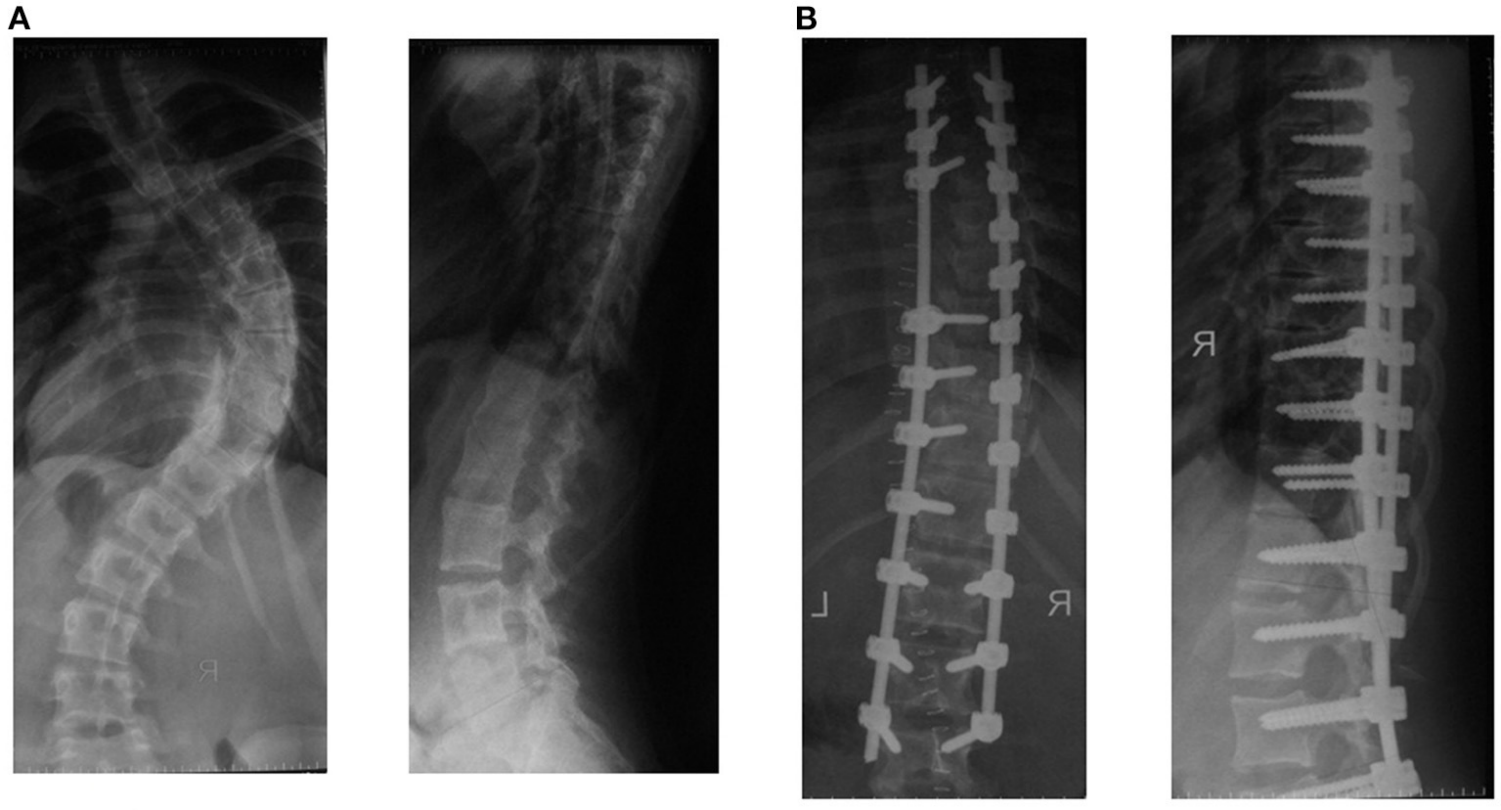
Spinal radiographs of patient B (16 years old). Idiopathic right thoracic scoliosis. **(A)** Preoperatively, the angle of deformity was 78° according to Cobb, the angle of kyphosis was 7°, and the angle of lordosis was 38°. **(B)** Postoperatively, the angle of scoliosis was 16° according to Cobb, the angle of kyphosis was 12°, and the angle of lordosis was 25°.

Various options for the correction of spinal deformity in children with idiopathic scoliosis of the thoracic spine exist. Using different levels and lengths for installation of transpedicular elements, the sequence of corrective manipulations as well as the use of the VCM system made it possible to individualize the approach and to correct all components of the deformity in three planes. A conducted study on surgical treatment in patients with idiopathic scoliosis showed that using transpedicular support elements through the whole curvature arch with VCM system, was significantly better in correction of the deformity (Figure 5) (scoliotic arch and rotation of the apical vertebra), compared to those in whom pedicle screws were not installed over two or more vertebrae from the concave side of the curvature (Figure 6). The kyphosis and lordosis were completely restored to their physiological values in all groups of patients.

## Data Availability Statement

The raw data supporting the conclusions of this article will be made available by the authors, without undue reservation.

## Ethics Statement

The studies involving human participants were reviewed and approved by University Medical Center. Written informed consent to participate in this study was provided by the participants' legal guardian/next of kin.

## Author Contributions

NN designed the study, collected the clinical data, and wrote the manuscript. SV collected the clinical data and contributed to revising the manuscript. All authors approved the submitted version.

## Conflict of Interest

The authors declare that the research was conducted in the absence of any commercial or financial relationships that could be construed as a potential conflict of interest.

## Publisher's Note

All claims expressed in this article are solely those of the authors and do not necessarily represent those of their affiliated organizations, or those of the publisher, the editors and the reviewers. Any product that may be evaluated in this article, or claim that may be made by its manufacturer, is not guaranteed or endorsed by the publisher.
